# Development and Characterization of Integrated Nano-Sensors for Organic Residues and pH Field Detection

**DOI:** 10.3390/s21175842

**Published:** 2021-08-30

**Authors:** Itamar Chajanovsky, Sarah Cohen, Giorgi Shtenberg, Ran Yosef Suckeveriene

**Affiliations:** 1 Department of Water Industry Engineering, Kinneret Academic College, Zemach 15132, Israel; itamar@kinneret.ac.il (I.C.); sarah.cohen00000@gmail.com (S.C.); 2 Institute of Agricultural Engineering, ARO, The Volcani Center, Bet Dagan 7505101, Israel; giorgi@volcani.agri.gov.il

**Keywords:** polyaniline, carbon-based nanocomposites, sensor, pH levels, organic residues

## Abstract

Meeting global water quality standards is a real challenge to ensure that food crops and livestock are fit for consumption, as well as for human health in general. A major hurdle affecting the detection of pollutants in water reservoirs is the lapse of time between the sampling moment and the availability of the laboratory-based results. Here, we report the preparation, characterization, and performance assessment of an innovative sensor for the rapid detection of organic residue levels and pH in water samples. The sensor is based on carbonaceous nanomaterials (CNMs) coated with an intrinsically conductive polymer, polyaniline (PANI). Inverse emulsion polymerizations of aniline in the presence of carbon nanotubes (CNTs) or graphene were prepared and confirmed by thermogravimetric analysis and high-resolution scanning electron microscopy. Aminophenol and phenol were used as proxies for organic residue detection. The PANI/CNM nanocomposites were used to fabricate thin-film sensors. Of all the CNMs, the smallest limit of detection (LOD) was achieved for multi-walled CNT (MWCNT) with a LOD of 9.6 ppb for aminophenol and a very high linearity of 0.997, with an average sensitivity of 2.3 kΩ/pH at an acid pH. This high sensor performance can be attributed to the high homogeneity of the PANI coating on the MWCNT surface.

## 1. Introduction

Water management worldwide falls into different categories, such as drinking water, irrigation, wastewater treatment, etc. The quality and regulation of water for agricultural use are a primary concern, since heavy metals, organic residues, and other pollutants can accumulate in the crops, affect their growth, and be consumed [1]. In Mainland China, for instance, half of all river waters are polluted and pose safety concerns for marine life [2], thus directly impacting food safety. For many years, the water supply for agricultural irrigation in most countries was derived from underground sources and rivers. However, overexploitation has led to major shortages, thus forcing policymakers to rethink methods of water supply management that are more sustainable and eco-friendly. In recent years, there have been greater efforts to collect and store rainwater in reservoirs, particularly in arid and semi-arid regions, given the massive variability in rainfall [3,4,5,6]. This water management approach has enormous potential, since it is low-cost, reasonably simple to implement, and increases crop productivity [7]. However, several studies have reported considerable amounts of pollutants in both the rainwater and runoffs collected in reservoirs, which require monitoring of the water quality before it can be put to any use. For example, the quality of rainwater from Polish reservoirs was shown to have a high salinity level, unacceptable phosphorus and chloride ion concentrations, and other pollutants in a lower load range, such as heavy metals [8]. Despite these lower percentages, heavy metals are known to be dangerous even at the trace level and can persist in the environment, and this may cause serious damage to both the environment and to human health even many years later [9,10]. Lee et al. [11] emphasized the need for reservoir maintenance, even if minimal, to mitigate microbiological and chemical pollution, for example, by installing flush filters to correct for the buildup of numerous contaminants that impact water quality as rainwater stagnates. Schets et al. [12] showed that the fecal and coliform contamination of harvested rainwater resulted in the growth of extremely harmful bacteria in reservoirs. Thus, overall, the amount of contaminants, including organic matter, bacteria, and heavy metals, are indicators of water quality and the potential risks to health.

The most common method of monitoring for the presence of pollutants and their concentration is through spectrometry analyses, which may also be coupled with fluorescence [13,14,15,16]. However, these systems are expensive and cannot deliver a rapid response in the field; that is, they cannot be used in real time [17].

Another popular technology is sensors, and, in particular, nanosensors, which are attracting increased interest for their ability to overcome the drawbacks described above while presenting several advantages, including rapidity, real-time analysis, portability, high sensitivity (detection trace levels), miniaturization, and simple preparation [18]. For these reasons, nanosensors are increasingly being used to detect and measure pollutants in water [19]. Shtenberg et al. [20] developed an optical biosensor based on horseradish peroxidase inhibition anchored onto nanostructured porous silicon that can detect and quantify a range of metallic ions in contaminated water.

In addition, advances in the sensitivity and selectivity of nanosensors—in particular, carbon nanotubes (CNT)-based sensors—have turned them into an innovative solution for the detection of analytes [21]. Wang et al. [22] described a glucose biosensor based on multi-wall CNTs (MWCNTs), glucose oxidase, and polypyrrole and reported a very high sensitivity of 2.33 nA/mM, along with high linearity. Savk et al. [23] described the simultaneous detection of three different types of organic matter, using a MWCNT-based sensor composed of embedded ZnNi bimetallic nanoalloys that also had excellent electrochemical properties.

Another promising alternative is optical pH-based sensors, because they can screen the pH of water in real time to determine whether the water sample is contaminated by pollutants. Dye indicators may be used to track the color changes based on differences in pH. Nevertheless, when using dyes, additional chemical or physical immobilizing steps need to be included in the sensor design [24,25,26]. Recently developed conductive pH-sensitive polymers make it possible to avoid using dyes.

The most efficient polymers today are considered to be intrinsically conducting polymers (CPs), because their outstanding electrical and optical properties lend themselves to a wide range of practical applications, including sensing [17,27]. Of these CPs, polyaniline (PANI) is the most frequent choice, given its ease of synthesis, low cost, good environmental stability, and ability to reverse its electric properties (doping) from an insulator to a metallic conductor through a protonation/deprotonation mechanism [28]. In an acidic medium, PANI is conductive in its protonated form, while, in a basic medium, PANI becomes an insulator (in its deprotonated form) [29,30]. One way to prepare PANI is by inverted emulsion polymerization [31,32,33], which has the advantage of overcoming the thermal and viscosity issues that can arise during the reaction, while maintaining its robust chemical properties [34]. Emulsion polymerization is a free-radical oil-in-water process comprising monomers, a dispersing medium, a surfactant, and an initiator in which the continuous aqueous phase contains the surfactant, and the organic phase contains the monomers. Polymerization takes place within spherical micelles created by the surfactant’s aggregation, which results in a stable colloidal dispersion, i.e., an emulsion. In the case of inverse emulsion polymerization, i.e., water-in-oil polymerization, the monomer is contained in the continuous phase [35].

However, PANI suffers from a lack of processability, which causes low solubility in the organic solvent. To overcome this problem, dopant molecules such as dodecylbenzene sulfonic acid (DBSA) [36,37] are incorporated along the PANI chain, thus increasing its solubility through charge-transfer doping [38].

Recently, a new class of materials made up of a combination of inorganic nanoparticles with organic polymeric compounds was introduced. CNTs and graphene are widely used as nanomaterials for sensors [39], and their association with PANI imparts synergetic properties to nanocomposit6es [40,41,42,43]. The outstanding structure and properties of CNTs (high stiffness, extraordinary flexibility, and tensile strength, along with high thermal and electrical conductivity) mean they can be used in a multitude of technological applications. One of the drawbacks of CNTs is their tendency to agglomerate, which decreases their surface area. However, several methods are currently available to prevent this, including using a surfactant to stabilize the CNTs, performing ultrasonication shear or in-situ polymerizing of monomers in the presence of CNT, or a combination of these methods [44]. Although the cost of neat graphene makes it less affordable for designing sensors, other variants have been used, such as reduced graphene oxide (rGO). Graphene oxide prepared from graphite is then chemically [45], thermally [46], or electrochemically reduced [47], leading to a lower oxygen content. One study reported that a calixarene-modified rGO-based electrochemical sensor detected different metal ions concurrently and demonstrated high sensitivity, selectivity, and reproducibility [48].

To respond to the shortcomings described above, the current paper describes an innovative rapid electrochemical pH-based nanosensor implementing a one-pot dye-free synthesis. Inverse emulsion polymerization of aniline is prepared in the presence of different grades of CNTs or graphene, along with DBSA as the dopant and polycaprolactone as the structural reinforcement within a toluene medium. The resulting dispersion is cast to produce the nanocomposite film. We report analyses of the pH sensitivity of the nanosensor that involved simultaneously measuring the pH with a pH-meter and conductivity with a two-probe apparatus. The findings show that, for the two organic residues (phenol and aminophenol) used, aminophenol presented higher sensitivity. These phenolic compounds are suitable indicators for water quality monitoring because their pH ranges from 5.0 to 8.0. The pH of wastewater is mostly between 6.0 and 7.5, since the acidification of the soil results in an increase in the pollutant load [49].

## 2. Materials and Methods

### 2.1. Materials

Distilled aniline (S.D. Fine-Chem Limited (SDFCL), Mumbai, India) was used as the monomer. DBSA was used as both the dopant and the surfactant without further purification (Aldrich, MA, USA). Two different types of polycaprolactones, namely PCL-1 (80 K, Melt Flow Index (MFI) 3 g/10 min, Sigma-Aldrich, UK) and PCL-2 (50 K, MFI 7 g/10 min, Tri Iso, CA, USA), were used as received. Four different types of carbon nanoparticles were used: (a) single-walled CNTs (SWCNTs, outer mean diameter of 1.6 ± 0.4 nm and length exceeding 5 µm, OCSiAl, Luxembourg), (b) multi-walled CNTs (MWCNTs, NC7000 with an average diameter of 9.5 nm and a length of 1.5 µm, Nanocel, Belgium), (c) reduced graphene oxide (rGO, Aldrich, platelet size of 1–3 µm, Graphenea, San Sebastián, Spain), and (d) graphene nanoplatelets (Graphene 300, Sigma-Aldrich, particle size below 2 µm, St. Louis, MO, USA). Ammonium peroxydisulfate (APS) initiator was used as received (Dayang Chem, Hangzhou, China). Toluene was used as a solvent (SDFCL, Mumbai, India). Ethanol (99.9%, Romical, Haifa, Israel) was used to wash the film after preparation and remove the dopant, aniline excess, and impurities.

### 2.2. Preparation of PANI/CNMs Nanocomposite Film

**Figure 1:**
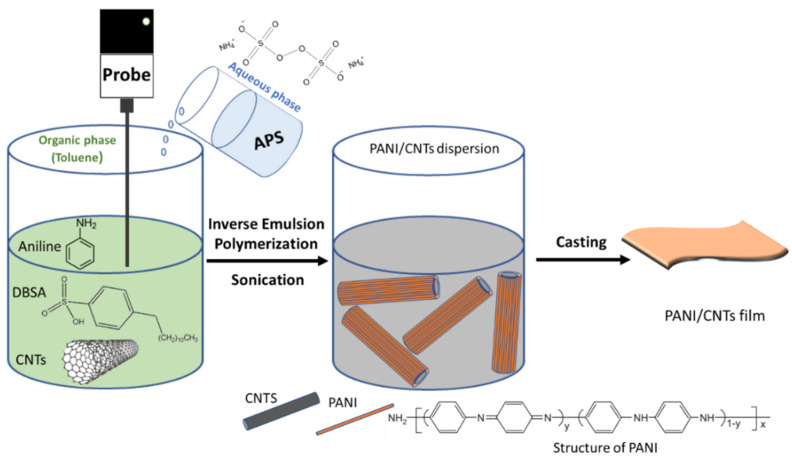
Inverse emulsion polymerization of aniline in the presence of CNTs for the fabrication of PANI/CNM composites.

An inverse emulsion polymerization of the aniline was carried out in the presence of four different types of CNMs and two different types of polycaprolactone under sonication, as described in Table 1. A typical preparation procedure is illustrated in Figure 1, as previously reported [33,50]. The organic phase was prepared as follows: 0.5 g of distilled aniline, 1.73 g of DBSA, 2 g of polycaprolactone (10% *w*/*v*), and CNMs at different concentrations (0.01, 0.02, 0.04, and 0.08% *w*/*v*) were placed in 19 mL toluene and sonicated for one minute. Note that the aniline polymerization took place without either CNMs or polycaprolactone and was conducted solely in the presence of MWCNTs, which are referred to henceforth as DBSA-doped PANI and PANI/MW composites, respectively. The aqueous phase was prepared by dissolving 0.31 g of APS in 1 mL of distilled water. The aqueous solution was added to the organic phase and sonicated for 5 min at 4 °C, using an ultrasonic liquid processor (750 Watt Sonicator, Sonics & Materials Inc., Newtown, CT, USA). Then, the resulting mixture was stored at 4 °C for 2 h to complete the polymerization.

The nanocomposite films were prepared on an MI-15 glossy paper substrate (Joliver, Afula, Israel). First, 2 mL of the resulting PANI/CNM dispersions was placed on the substrate, and a thin film was prepared by using coating rods (K-rods, 4 µm wet thickness, RK Print, Royston, UK). The film was left in the fume hood to dry, followed by ethanol rinsing to remove impurities.

### 2.3. Characterization

The morphology of the PANI/CNM nanocomposites was investigated with a High-Resolution Scanning Electron Microscope (HR-SEM, Carl Zeiss Ultra Plus, Zeiss, Jena, Germany), equipped with a high-resolution field emission gun. The samples were freeze-fractured and sputtered with gold prior to observation, and then they were examined by using an accelerating voltage of 4 keV.

The thermal stability of the PANI/CNM nanocomposites was determined by thermogravimetric analysis (TGA), using a TA Instruments Q5000 Thermal Gravimetric Analyzer (TA Instruments, New Castle, DE USA). The temperature range was 25–800 °C, at a heating rate of 10 °C/min, while monitoring for weight loss as a function of temperature. The analysis was conducted under a nitrogen atmosphere, at a flow rate of 25 mL/min.

The electrochemical and sensitivity performances of the sensor were examined by using a pH-meter (Adwa-Ad8000, Adwa Instruments, Szeged, Hungry) and a multimeter (KEITHLEY 2000, Tektronix, Oldbury, UK) equipped with a two-probe system. Two types of solutions, 1 M sodium hydroxide (NaOH) and 1 M hydrochloric acid (HCl) (Carlo Erba Reagents, Val de Reuil, France), were added to the water solution to monitor the resistivity as a function of pH. In addition, phenol (Alfa Aeser, Heysham, UK) and aminophenol (Sigma-Aldrich, St. Louis, MO, USA) were used as organic residue templates to evaluate the conductivity change as a function of pH. In general, electrical conductivity is defined as the inverse of the resistivity value. In the case of thin films, this is the sheet resistance (Ω/□ or Ω per square) measured with a four-point probe, as expressed in Equation (1):

(1) $$R\;\left(\Omega \right)=\frac{\rho \;\left(\Omega .\mathrm{cm}\right)\;\times \;L\;\left(\mathrm{cm}\right)}{t\;\left(\mathrm{cm}\right)\;\times \;w\;\left(\mathrm{cm}\right)\;}\;=\;\frac{\mathrm{Rsh}\times \;L}{w}$$

Equation (1): Relationship between the sheet resistance and the electrical resistivity, where R is the resistance; ρ is the bulk resistivity; and L, w, and t are the length, width, and thickness of the resistor, respectively. The sheet resistance, R_sh_, is expressed as ρ/t [51].

## 3. Results

### 3.1. Polymerization of Polyaniline in the Presence of CNTs

#### 3.1.1. Color Change of PANI Film

**Figure 2:**
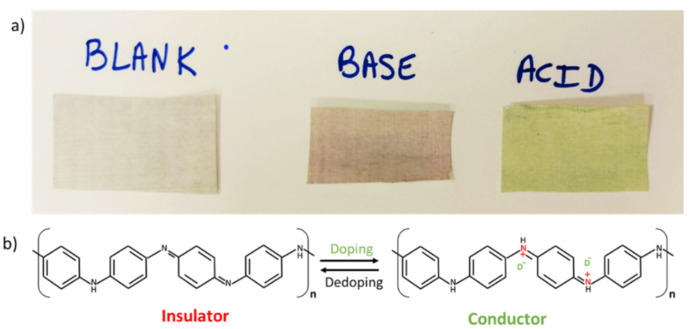
(**a**) Illustration of color changes in the PANI/MW/P1 films at (right) pH = 1 and (middle) pH = 13. (**b**) Schematic structure of PANI depicting the doping/de-doping process.

Figure 2a illustrates the PANI color changes in the PANI/MW/P1 films at acidic and basic pHs of 1 and 13, respectively. As described in the literature [40], PANI possesses three different oxidation states. The half-oxidized state is known as the emeraldine base. It is capable of switching from the insulating state to the conductive state by protonating the imine nitrogen groups of the PANI backbone by strong acids (Figure 2b). This phenomenon is usually typified by a change in the PANI color due to oxidation. The film that was originally white (blank) turned green (protonated, doped form) or reddish (deprotonated, de-doped form) at acidic and basic pHs, respectively [52]. The PANI/CNM films can hence potentially be used as an electrochemical pH-based sensor.

The polymerization of aniline in the presence of different types of CNTs and graphene was examined by TGA and HR-SEM. The characteristic Fourier Transform Infrared Spectroscopy (FTIR) peaks can be seen in Supplementary Materials Table S1.

#### 3.1.2. HR-SEM Analysis

**Figure 3:**
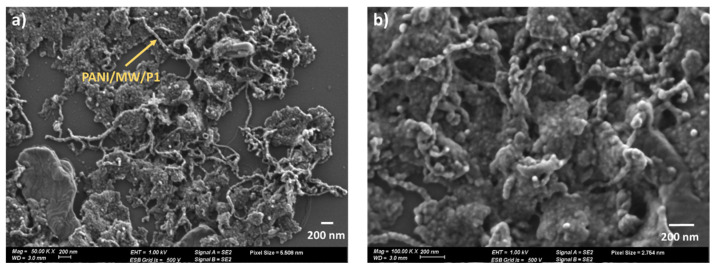
HR-SEM images of the PANI/MW/P1 at a magnification of (**a**) 50 and (**b**) 100 KX.

The morphology of the functionalized MWCNTs polymerized in toluene is depicted in Figure 3. The HR-SEM images show the successful coating of NC7000 with PANI, labeled as PANI/MW/P1. The elongated entangled chains depict the polymerization of aniline over the walls of MWCNTs. Previously we showed that PANI was covalently attached to the surface of CNTs [50,53]. PANI wrapping the nanotubes resulted in a dense network; some PANI that were unattached to the CNT surface accumulated on the silicon wafer’s surface, generating large agglomerates.

#### 3.1.3. TGA Analysis

**Figure 4:**
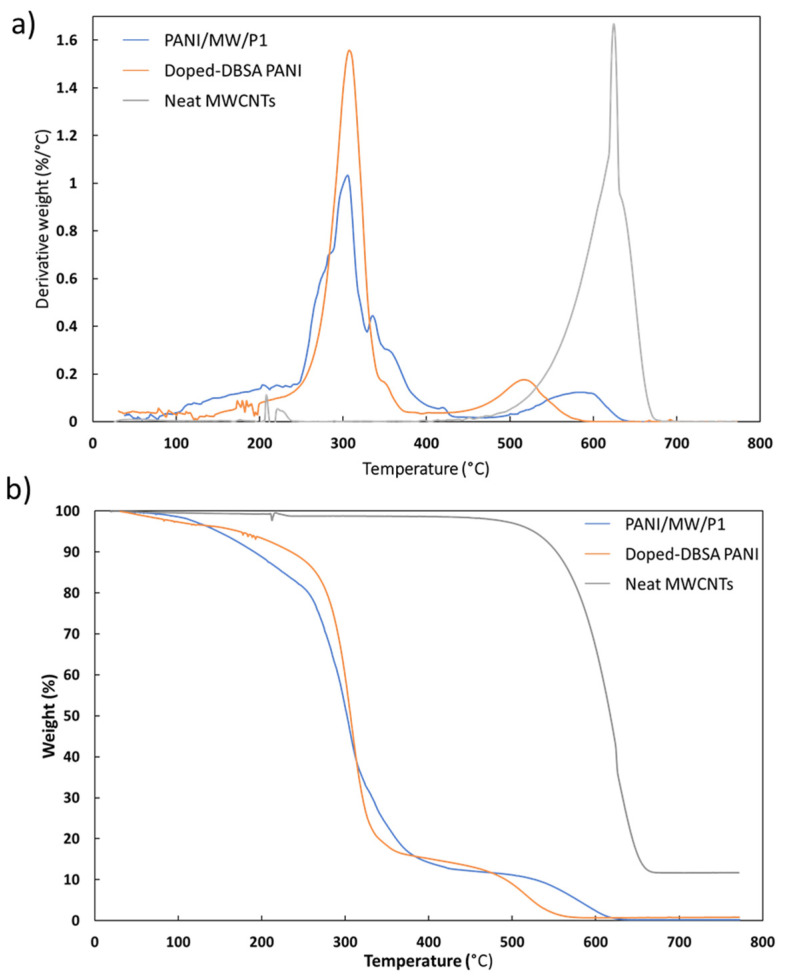
(**a**) DTG and (**b**) TGA thermograms of neat MWCNTs (gray line), doped-DBSA PANI (orange line), and PANI/MW/P1 (blue line).

Figure 4 depicts the TGA and DTG (first derivative) thermograms of pristine MWCNTs (gray line), DBSA-doped PANI (orange line) and PANI/MW/P1 nanocomposite (blue line). The other PANI/CNM nanocomposites exhibited similar behavior (Supplementary Materials Figure S1). The DTG curve shows that the pristine MWCNTs had a single weight loss, which occurred at 625 °C. Doped-DBSA PANI underwent a two-stage degradation, whereas the PANI/MW/P1 nanocomposite evidenced four weight losses. The weight loss in the temperature range of 270–330 °C can be attributed to the thermal decomposition of DBSA molecules that are in interaction with the PANI chains, whereas the second transition occurring at ~517 °C can be attributed to the degradation of PANI itself [54]. The DTG curve of the PANI/MW/P1 nanocomposite presented two new peaks at ~335 and 422 °C, which can be attributed to the bonded dopant and the PCL-1 decomposition, respectively. The fourth thermal transition, whose peak was located at ~590 °C, may be due to the PANI backbone and MWCNT degradation. The other PANI/CNM nanocomposites showed a decrease in thermal stability; the temperature of peak in the range of 270–330 °C decreased slightly compared to DBSA-doped PANI. It has been suggested [55] that the interactions between PANI and CNMs are weaker, thus resulting in poorer homogeneity. The addition of the MWCNTs yielded a more uniform coating of PANI onto the carbon surface, which improved the thermal stability of the nanocomposite.

### 3.2. Sensor Performance

#### 3.2.1. Effects of the Reinforcing Additive

In order to enhance the intrinsic conductivity of PANI, two electrically conductive polycaprolactones, PCL-1 and PCL-2, were added as reinforcement to the MWCNT-based nanocomposites. Note that the molecular weight of PCL-1 is higher. A 10 wt.% loading was incorporated in the nanocomposites, and their conductivities were calculated according to Equation (1), where the dimension of the resistor was 4 cm × 1 cm. The electrical conductivities of the PANI/MW/P1 and PANI/MW/P2 films were 4.76 × 10^−4^ S/cm and 3.64 × 10^−4^ S/cm, respectively. Regardless of the type of polycaprolactone, the addition of reinforcement considerably improved conductivity relative to the pure PANI (blank) film, which was 2.09 × 10^−4^ S/cm. The analysis of the MWCNT content increased in the 0.02–0.08 wt.% range and showed similar behavior (data summarized in Supplementary Materials Table S2), except for the 0.04 wt.% MWCNT samples, where higher electrical conductivity was found for PCL-2. Hence, increasing the additive’s molecular weight led to a significant increase in the conductivity of the PANI films. In addition, because the lowest MWCNT content yielded the highest electrical conductivity, only the effect of the addition of PCL-1 and 0.01 wt.% CNMs on sensor performance was examined for the other PANI/CNM nanocomposites.

**Figure 5:**
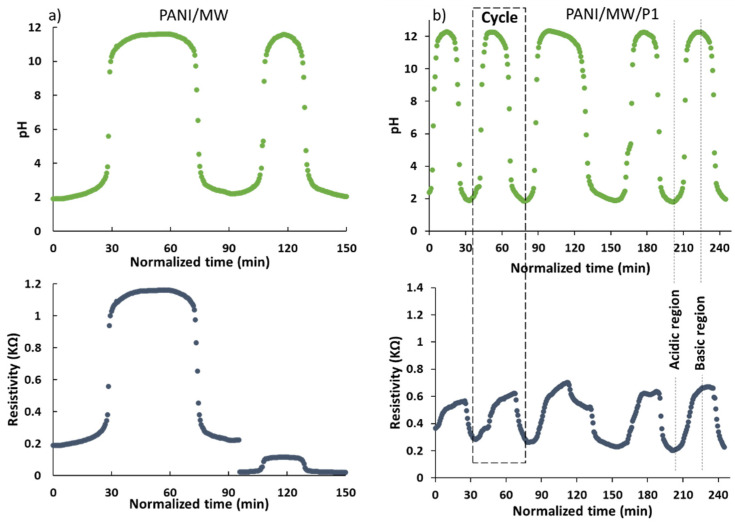
Changes in resistivity as a function of pH over time in the (**a**) PANI/MW and (**b**) PANI/MW/P1 samples.

Figure 5 depicts the resistivity response of the PANI/MW and PANI/MW/P1 sensors to pH changes in a water solution over time. The sensors underwent initial activation prior to the beginning of measurement, hence normalizing the duration. Each cycle was triggered by adding the HCl solution, followed by NaOH, resulting in a continuous alternation of acidic and basic pH. It is clear that, without reinforcement, the sensor performances were low, since the resistivity dropped brutally during the second cycle, showing the non-reproducibility of the sensor in the long-term. This resistivity drop may be explained by the changes in the electrical behavior of the PANI which became insulating. In contrast, the incorporation of PCL-1 considerably enhanced stability, because, at each new cycle, the initial resistivity value was almost fully regained, demonstrating the apparent role of PCL-1 as reinforcement in the sensor. Similar behavior was observed in the PANI/MW/P2 film; however, it exhibited lower conductivity than the PCL-1. This may be explained by the presence of a larger number of carboxylic acid groups within the PCL-1 backbone chain that may have acted as an additional dopant, thus providing more counter-anions along the PANI chains. The solubility, as well as the electrical conductivity of PANI, was therefore enhanced as a result of the increase in the number of cation charge carriers NH^+^ on the PANI chains [56]. The performances of the sensor still remained very accurate, with little or no loss of efficiency even after four hours of runtime, thus demonstrating the non-deterioration and reliability of the sensor with time.

#### 3.2.2. Sensor Performances as a Function of the Type of Carbonaceous Nanomaterials

**Figure 6:**
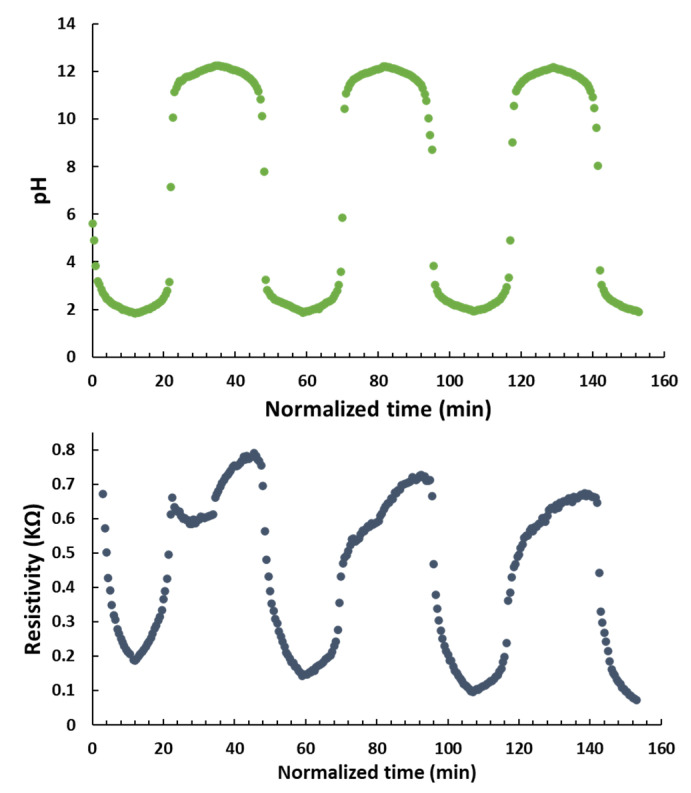
Changes in resistivity as a function of pH over time in the PANI/rGO/P1 sample.

The chemical structure of each CNM played a significant role in the performance of the sensors. As a function of structural dimensions, the sensor’s effectiveness can be affected in terms of conductivity, stability, reproducibility, and sensitivity. As seen in Table 1, all the 0.01 wt.% CNTs samples exhibited higher electrical conductivity than the pure PANI. Adding SWCNTs enhanced the conductivity by one order of magnitude, reaching 1.32 × 10^−3^ S/cm. An improvement was also observed for the graphene-based films, with graphene 300 showing better conductivity than rGO, i.e., 2.63 × 10^−4^ S/cm and 2.25 × 10^−4^ S/cm, respectively. Each sample presented an order of magnitude greater than or equal to 10^−4^, which indicates that the films were suitable semiconductors [57]. Although the two-dimensional (2D) structure of the graphene sheets provided a larger surface area than the one-dimensional (1D) structure of CNTs, the probability of restacking was greater in the 2D structure so that the real available surface decreased [58]. In addition, as shown in Figure 6, the PANI/rGO/P1 sensor exhibited slightly lower stability than the PANI/MW/P1 sensor. At each cycle, the resistivity value at the lowest and highest pH point was not fully recovered after several consecutive cycles. A systematic ~0.06 Ω/□ decrease was apparent at the start of each new cycle. The 1D structure of CNTs presented a higher distribution surface than the 2D structure of rGO, resulting in better PANI wrapping to the CNTs surface. The steric hindrance generated by PANI coating prevented the CNTs from re-aggregating to each other, thus avoiding cluster formation and allowing the sensor to preserve its excellent performance. The synergic effect of the high surface area of CNTs and high electrical conductivity of the PANI contributed to the high capacity of the sensor as well [44].

#### 3.2.3. Sensor Sensitivity for Detecting Phenol and Aminophenol

**Figure 7:**
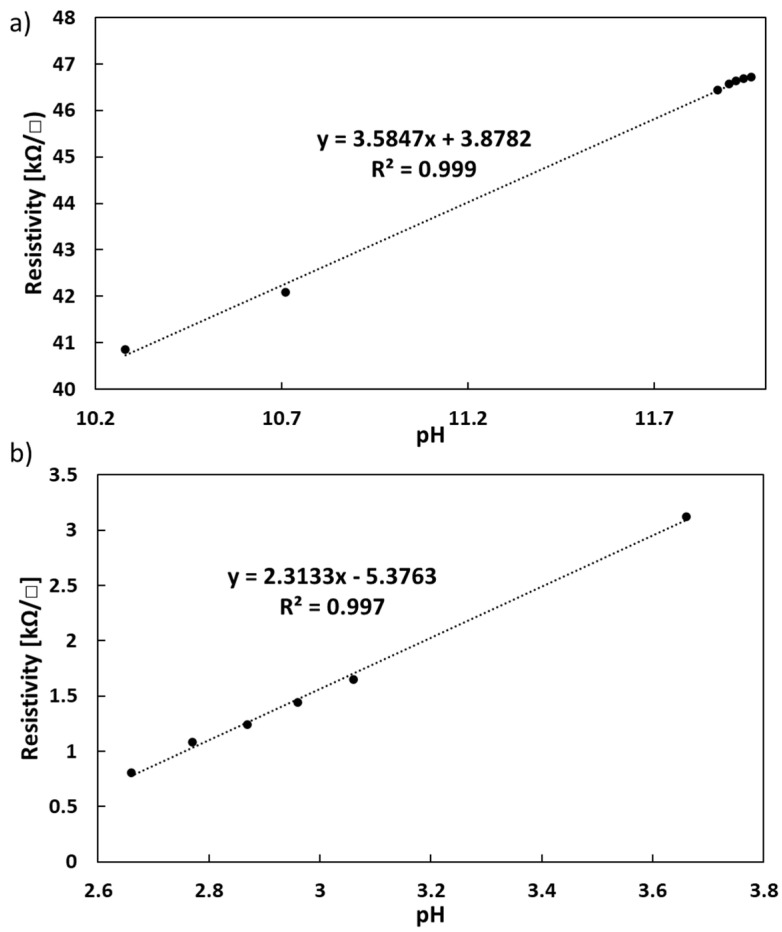
Calibration curve for pH of PANI/MW/P1 film at (**a**) basic and (**b**) acidic pH levels.

PANI is an intrinsically conducting polymer that is known to be strongly dependent on pH. The more the pH decreases, the more the electrical conductivity increases. This can be explained by the higher mobility of π-electrons along the PANI backbone at acidic levels, due to a higher doping level [59]. Figure 7 shows the calibration curve of the PANI/MW/P1, representing the basic and acidic pH response ranges. The film’s resistivity is clearly dependent upon pH values, because each small increment of an acidic or basic solution immediately altered the sheet resistance. The graph shows that the resistivity was lower at acidic pHs (0.5–3 kΩ/□) than at basic pHs (41–47 kΩ/□), confirming that conductivity was enhanced when the pH decreased. The sensor also showed high sensitivity, with a high linearity of 99.7% and 99.9% and an average sensitivity of 2.3 and 3.6 kΩ/pH, at acidic and basic pHs, respectively.

**Figure 8:**
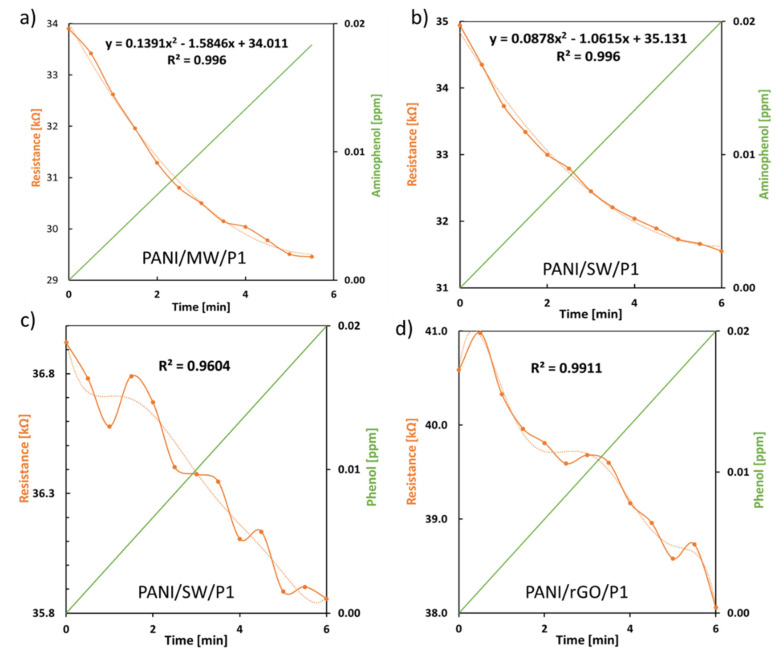
Electrochemical measurements of the sheet resistance of (**a**) PANI/MW/P1, (**b**,**c**) PANI/SW/P1, and (**d**) PANI/rGO/P1 sensors as a function of the (top) aminophenol and (bottom) phenol concentration.

Next, the sensor’s inherent sensitivity to phenol and aminophenol as organic residues was tested by monitoring the resistivity change as a function of the analyte concentration over time for the sensors showing the highest and lowest electrical conductivity. Figure 8a,b illustrate the resistivity response plotted against the aminophenol concentration of the PANI/MW/P1 and PANI/SW/P1 sensors, respectively. As the concentration increased, the electrical conductivity also increased. From the very first drop of aminophenol, there was an immediate change in the resistivity that constantly decreased, which characterized the fast real-time response towards the analyte trace level. The plot of the resistivity vs. the aminophenol concentration exhibits a non-linear decrease as the analyte concentration increases, which was a good fit with a second-order polynomial [60,61]. This may be due to the decrease in the available sensing layer that could have interacted with the analyte. The PANI/SW/P1 (Figure 8c) and PANI/rGO/P1 sensors (Figure 8d) also exhibited a fast response to phenol, since the resistivity decreased as the concentration increased within a few minutes. In this case, the non-linearity of the curve appeared to fit a sixth-order polynomial. In all experiments, as the analyte concentration increased, the pH of the solution only varied very slightly, i.e., ~0.02 at each increment. For the two analytes, the concentration corresponded to a pH ranging from ~5.4 to 6.4, thus closely reflecting the pH of wastewater. Hence, the sensor showed high sensitivity and could detect analytes at trace levels, despite the non-linearity. This is likely due to the fact that the incorporation of CNMs resulted in an increase of the specific surface area and that the addition of polycaprolactone increased the electrical conductivity hence leading to enhanced sensitivity.

#### 3.2.4. Limits of Detection

**Figure 9:**
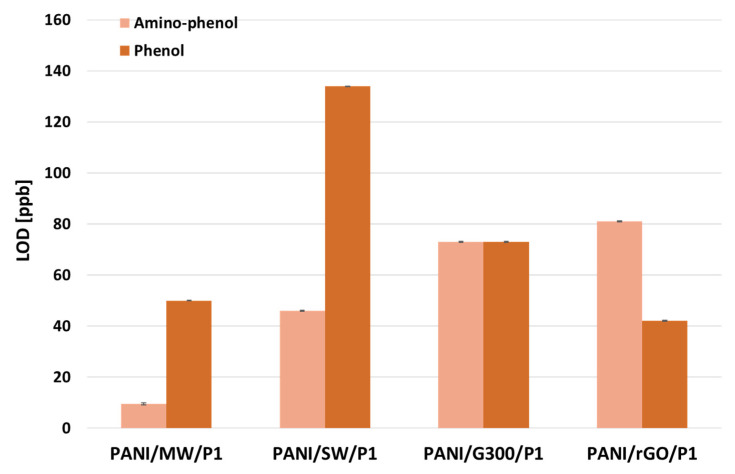
Histograms showing the LOD of the PANI sensor as a function of carbon type of aminophenol and phenol.

The limits of the sensor for detecting low concentrations of the aminophenol and phenol solutions were examined (Figure 9). Two parameters are typically utilized to assess the sensitivity and detection threshold of a sensor: the limit of detection (LOD) and the quantification (LOQ), which are defined as the lowest concentration of an analyte in a sample that can be detected and the lowest concentration of analyte in a sample that can be quantitatively determined with suitable precision and accuracy, respectively [62]. By definition, LOQ is always higher than LOD. According to the ICH (International Conference on Harmonization), LOD and LOQ can be calculated as shown in Equation (2):

(2) $$LOD,\;LOQ=\frac{F\;\times SD\left[Blank\right]}{b}$$

Equation (2): limit of detection (*LOD*) and limit of quantification (*LOQ*), where *F* is equal to 3.3 and 10, respectively. *SD* is the standard deviation of the blank solution (without an analyte; at least three measurements), and *b* (sensitivity) is the slope of the regression line [62].

Each film showed a very high sensitivity in the range of ppb, regardless of the organic residues. Overall, the films showed a higher sensitivity to aminophenol than phenol, resulting in a smaller LOD. For the detection of aminophenol, the CNT-based films exhibited the smallest LODs and LOQs, i.e., 9.6 and 29 ppb for PANI/MW/P1, and 46 and 140 ppb for PANI/SW/P1, respectively. Although the sensitivity response to aminophenol was quite similar to what was observed for the MWCNTs, the LOD value for PANI/SW/P1 was higher. On the other hand, the PANI/rGO/P1 sensor showed reverse sensitivity and was found to be the most sensitive to the detection of phenol with a LOD of 42 ppb, although the LOD for MWCNTs was relatively similar (50 ppb). Interestingly, SWCNTs showed the greatest LOD with phenol. The high PANI/MW/P1 sensor performances support the assumption that PANI was distributed more uniformly over the surface of MWCNTs. The PANI/G300/P1 sensor displayed the same sensitivity for both organic residues, i.e., 73 ppb, which was slightly lower than aminophenol LOD (81 ppb) for rGO, but much higher than phenol. It has been suggested that the oxygen defects present in rGO acted as chemically active sites, which enable the phenol selectivity to be enhanced [63].

## 4. Conclusions

A straightforward electrochemical pH-based nanosensor was designed for the detection of organic residues. The successful and rapid inverse emulsion polymerization of aniline in the presence of carbon contributed to facilitating the sensor design. The polycaprolactone incorporation as reinforcement considerably improved the conductivity of PANI at low CNT content, thus making it easy to measure the sheet resistivity of the films as a function of the pH. The lowest LOD was for sensors based on CNTs as compared to graphene, due to their larger available surface area, which resulted in an enhancement of the solubility of PANI in toluene. Aminophenol showed very high sensitivity of the sensor in the range of ppb at 9.6 ppb for the PANIMW/P1 sample.

To further improve electrical conductivity, we created nanocomposite fibers by electrospinning. This structure is currently being characterized. The preliminary results are promising, since the obtained LOD for detecting aminophenol became even smaller. Future work will concentrate on this topic.

## Figures and Tables

**Table 1:** Preparation of different nanocomposites and their electrical conductivity.

Samples	MWCNT (0.01 wt.%)	PCL-1 (10 wt.%)	PCL-2 (10 wt.%)	Electrical Conductivity (S/cm) ^1^
DBSA-doped PANI (Blank)	0.002 g			2.09 × 10^−4^
PANI/MW	0.002 g			2.20 × 10^−4^
PANI/MW/P1	0.002 g	2 g		4.76 × 10^−4^
PANI/MW/P2	0.002 g		2 g	3.64 × 10^−4^
	**SWCNT**	**PCL-1**		
PANI/SW/P1	0.002 g	2 g		1.32 × 10^−3^
	**Graphene 300**	**PCL-1**		
PANI/G300/P1	0.002 g	2 g		2.63 × 10^−4^
	**rGO**	**PCL-1**		
PANI/rGO/P1	0.002 g	2 g		2.25 × 10^−4^

^1^ The electrical conductivity was measured with a four-point probe (see Equation (1)). Polyaniline (PANI), dodecylbenzene sulfonic acid (DBSA), polycaprolactone (PCL), multi-wall CNT (MWCNT), single-wall CNT (SWCNT), reduced graphene oxide (rGO).

## Supplementary Materials

The following are available online at https://www.mdpi.com/article/10.3390/s21175842/s1. Table S1. Characteristic FTIR peaks of the DBSA-doped PANI and PANI/MW/P1 samples. Table S2. Summary of the electrical conductivity of the PANI/MW/P1 and PANI/MW/P2 samples in a range of 0.02–0.08 wt.% for MWCNTs. Figure S1. DTG (a and c): DTG, and TGA (b and d) thermograms of (Left) PANI/G300/P1 and (Right) PANI/SW/P1.
