# Supplementary material for: Development and Characterization of Integrated Nano-Sensors for Organic Residues and pH Field Detection
Source: Sensors (Basel). 2021 Aug 30;21(17):5842. doi: 10.3390/s21175842 (PMC8434280; doi:10.3390/s21175842)
Supplement: Supplementary file 1 [file sensors-21-05842-s001.zip › sensors-1353601-supplementary.pdf]

Supplementary Materials

Development and Characterization of Integrated Nano-Sensors for Organic Residues and pH Field Detection

Itamar Chajanovsky [1], Sarah Cohen [1], Giorgi Shtenberg [2] and R. Y. Suckeveriene [1,*]

[1] Department of Water Industry Engineering, Kinneret Academic College, Zemach 15132, Israel
[2] Institute of Agricultural Engineering, ARO, The Volcani Center, Bet Dagan 7505101, Israel

1. FTIR analysis

The Fourier Transform Infrared (FTIR) spectra of the PANI/CNM nanocomposites were in a range of 600 to 4000 cm^{-1} obtained on a Thermo 6700 FTIR equipped with a Smart iTR diamond ATR.

Table S1. Characteristic FTIR peaks of the DBSA-doped PANI and PANI/MW/P1 samples.

DBSA-doped PANI	PANI/MW/P1	Attribution
Wavenumber [cm^{-1}]		
3427	3427	N-H stretching vibration from emeraldine base salt [1]
	3204	O-H stretching (PCL)
	2954	C-H stretching (PCL)
2926	2920	C-H_2 stretching vibrations (DBSA)
2857	2851	C-H_3 stretching vibrations (DBSA)
1497	1497	C=C from quinoid rings [2] [3]
1460	1454	C=C from benzenoid rings
1417	1408	C-N stretching in quinonoid-benzenoid [4] [5]
1394	1377	C-N stretching in quinonoid-benzenoid
1084	1058	C-H bond deformation vibration (PANI) [6] [2]
1033	1035	SO_3^- group (DBSA) [1] [2] [7]
831	829	C-H out-of-plane deformation vibration (PANI) [8]
666	666	S=O stretching (DBSA)

2. TGA analysis

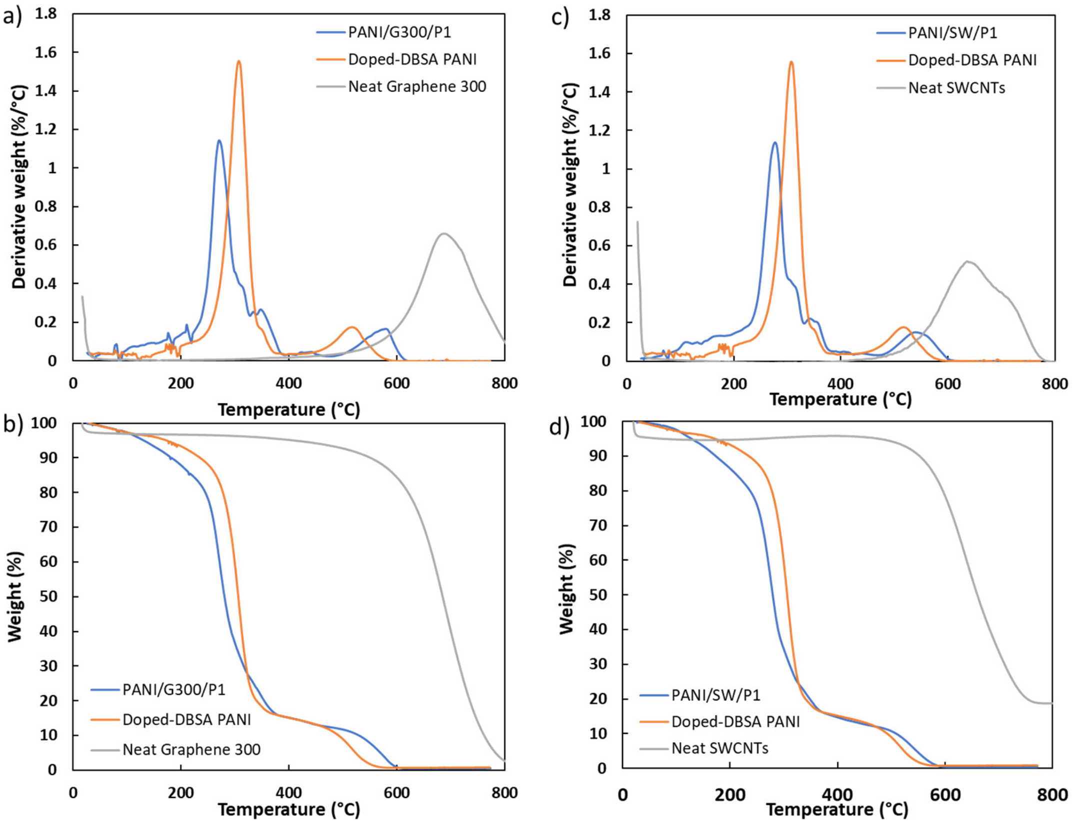

Figure S1. DTG (a and c): DTG, and TGA (b and d) thermograms of (Left) PANI/G300/P1 and (Right) PANI/SW/P1.

3. Electrochemical measurements

Table S2. Summary of the electrical conductivity of the PANI/MW/P1 and PANI/MW/P2 samples in a range of 0.02 – 0.08 wt.% for MWCNTs.

MWCNT content	0.02 wt.%	0.04 wt.%	0.08 wt.%
		Electrical conductivity [S/cm]	
10 wt.% PCL-1	3.23×10^{-4}	3.08×10^{-4}	2.60×10^{-4}
10 wt.% PCL-2	3.03×10^{-4}	3.45×10^{-4}	2.11×10^{-4}

References

[1] Yadav A, Kumar J, Shahabuddin M, Agarwal A, Saini P. Improved ammonia sensing by solution processed dodecyl benzene sulfonic acid doped polyaniline nanorod networks. IEEE Access 2019;7:139571–9. https://doi.org/10.1109/ACCESS.2019.2942361.

[2] Kumar J, Shahabuddin M, Singh A, Singh SP, Saini P, Dhawan SK, et al. Highly sensitive chemo-resistive ammonia sensor based on dodecyl benzene sulfonic acid doped polyaniline thin film. Sci Adv Mater 2015;7:518–25. https://doi.org/10.1166/sam.2015.2000.

[3] R. Y. Suckeveriene, E. Zelikman, G. Mechrez, A. Tzur, I. Frisman, Y. Cohen MN. Synthesis of Hybrid Polyaniline/Carbon Nanotube Nanocomposites by Dynamic Interfacial Inverse Emulsion Polymerization Under Sonication. J Appl Polym Sci 2010;120:2658–67. https://doi.org/10.1002/app.

[4] Regueira R, Suckeveriene RY, Brook I, Mechrez G, Tchoudakov R, Narkis M. Investigation of the Electro-Mechanical Behavior of Hybrid Polyaniline/Graphene Nanocomposites Fabricated by Dynamic Interfacial Inverse Emulsion Polymerization. Graphene 2015;04:7–19. https://doi.org/10.4236/graphene.2015.41002.

[5] Rao PS, Subrahmanya S, Sathyanarayana DN. Inverse emulsion polymerization: A new route for the synthesis of conducting polyaniline. Synth Met 2002;128:311–6. https://doi.org/10.1016/S0379-6779(02)00016-4.

[6] Nguyen VH, Lamiel C, Kharismadewi D, Tran VC, Shim JJ. Covalently bonded reduced graphene oxide/polyaniline composite for electrochemical sensors and capacitors. J Electroanal Chem 2015;758:148–55. https://doi.org/10.1016/j.jelechem.2015.10.023.

[7] Han D, Chu Y, Yang L, Liu Y, Lv Z. Reversed micelle polymerization: A new route for the synthesis of DBSA-polyaniline nanoparticles. Colloids Surfaces A Physicochem Eng Asp 2005;259:179–87. https://doi.org/10.1016/j.colsurfa.2005.02.017.
[8] Bachhav SG, Patil DR. Synthesis and Characterization of Polyaniline-Multiwalled Carbon Nanotube Nanocomposites and Its Electrical Percolation Behavior. Am J Mater Sci 2015;5:90–5. https://doi.org/10.5923/j.materials.20150504.03.
